# Clinical Outcomes of Biodegradable versus Durable Polymer Drug Eluting Stents in Rotational Atherectomy: Results from ROCK Registry

**DOI:** 10.3390/jcm11216251

**Published:** 2022-10-23

**Authors:** Kyung An Kim, Sung-Ho Her, Kyusup Lee, Ik Jun Choi, Jae-Hwan Lee, Jang Hoon Lee, Sang Rok Lee, Pil Hyung Lee, Seung-Whan Lee, Ki Dong Yoo, Su Nam Lee, Won Young Jang, Donggyu Moon, Keon-Woong Moon, Kyeong Ho Yun, Hyun-Jong Lee

**Affiliations:** 1 Department of Cardiology, Seoul St. Mary’s Hospital, College of Medicine, The Catholic University of Korea, Seoul 65091, Korea; 2 Department of Cardiology, St. Vincent’s Hospital, College of Medicine, The Catholic University of Korea, Seoul 65091, Korea; 3 Department of Cardiology, Daejeon St. Mary’s Hospital, College of Medicine, The Catholic University of Korea, Seoul 65091, Korea; 4 Department of Cardiology, Incheon St. Mary’s Hospital, College of Medicine, The Catholic University of Korea, Incheon 21431, Korea; 5 Department of Cardiology, Chungnam National University Sejong Hospital, College of Medicine, Chungnam National University, Sejong 34134, Korea; 6 Department of Internal Medicine, Kyungpook National University Hospital, College of Medicine, Kyungpook National University, Daegu 41944, Korea; 7 Department of Cardiology, Chonbuk National University Hospital, Jeonju 54907, Korea; 8 Department of Cardiology, Asan Medical Center, College of Medicine, University of Ulsan, Seoul 05505, Korea; 9 Department of Cardiovascular Medicine, Regional Cardiocerebrovascular Center, Wonkwang University Hospital, Iksan 54538, Korea; 10 Department of Internal Medicine, Sejong General Hospital, Bucheon 14754, Korea

**Keywords:** calcified coronary lesion, rotational atherectomy, second generation drug eluting stent, biodegradable polymer, durable polymer

## Abstract

Background: The aim of this study was to compare the clinical outcomes of biodegradable polymer (BP) versus durable polymer (DP) drug eluting stents (DES) in patients with calcified coronary lesions who underwent rotational atherectomy (RA) and percutaneous coronary intervention (PCI). Methods: This study was based on a multicenter registry which enrolled patients with calcified coronary artery disease who received PCI using RA during between January 2010 and October 2019 from 9 tertiary centers in Korea. The primary outcome was 3-year all-cause mortality, and the secondary outcomes were cardiovascular death and target-lesion failure. Results: A total of 540 patients who underwent PCI using RA were enrolled with a follow-up period of median 16.1 months. From this registry, 272 patients with PCI using DP-DES and 238 patients with BP-SGDES were selected for analysis. PCI with BP-DES was associated with decreased all-cause mortality after propensity score matching (HR 0.414, CI 0.174–0.988) and multivariate Cox regression analysis (HR 0.458, HR 0.224–0.940). BP-DES was also associated with decreased cardiovascular mortality, but there was no difference in TLF between the two groups. Conclusions: BP-DES were associated with favorable outcomes compared to DP-DES in patients undergoing PCI using RA for calcified coronary lesions.

## 1. Introduction

Severely calcified coronary lesions are one of the most difficult challenges to the intervention cardiologist. Heavy calcium deposition increases the complexity of the procedure and the likelihood of procedural failure by interfering with lesion preparation and balloon expansion, making device delivery difficult, and limiting final stent expansion [1]. Additionally, they are associated with a high frequency of restenosis and need for repeat revascularization, which is likely to adversely impact both the short- and long-term outcomes of coronary artery disease [2,3].

Rotational atherectomy (RA) is one of the preferred methods used during PCI for heavily calcified coronary lesions. It modifies calcified plaques leading to lumen enlargement and better stent expansion [4]. Early applications of RA either alone [5] or using bare metal stents (BMS) [6] were associated with high rates of restenosis and repeat revascularization. The introduction of drug-eluting stents (DES) in combination with RA has been shown to be safe and associated with better clinical outcomes compared to BMS [7,8,9]. Second generation DES (SGDES) have been a further improvement on the first-generation stents in terms of safety and efficacy when used after RA [10,11,12]. Additionally, there is accumulating evidence that newer ultrathin stents and biodegradable polymers are associated with more favorable results in patients undergoing PCI compared to thicker strut SGDES and durable polymers [13,14,15,16].

However, it is yet uncertain whether biodegradable polymer DES (BP-DES) are superior to durable polymer DES (DP-DES) in heavily calcified lesions requiring RA. In this study, our objective was to find out if there were clinical differences in BP-DES versus DP-DES in patients who underwent coronary stent implantation after RA for heavily calcified lesions.

## 2. Methods

### 2.1. Study Design and Population

**Figure 1:**
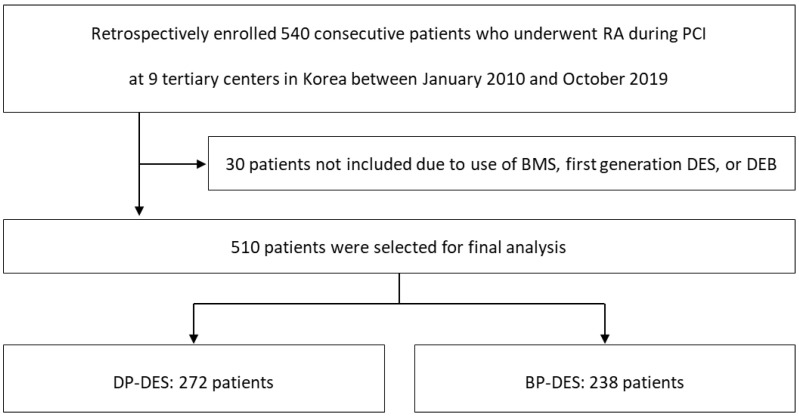
Study Population Flow Chart. RA, rotational atherectomy; PCI, percutaneous coronary intervention; BMS, bare metal stent; DES, drug eluting stent; DEB, drug eluting balloon; DP, durable polymer; BP; biodegradable polymer.

This study was based on the Rotational atherectomy in Calcified lesions in Korea (ROCK) registry. Details of the registry have been published elsewhere [17]. In brief, 540 patients who underwent PCI using RA due to calcified CAD between January 2010 and October 2019 at 9 tertiary centers in Korea were retrospectively enrolled and analyzed. The median follow-up period was 16.1 months (interquartile range 8.8, 38.4 months). For the purposes of the current analysis, 30 patients were dropped out due to use of BMS, first generation DES, and drug eluting balloons. The remaining 510 patients were divided into two groups according to the durability of the DES polymer used during the procedure. (Figure 1).

Data including demographic, clinical, angiographic and procedural characteristics were collected at each site using a standardized report form. Approval was given by the local ethics committee of each hospital. The study protocol was approved by the Institutional Review Board of each institution and is in accordance with the Declaration of Helsinki.

### 2.2. Stent Selection

The following stents in the ROCK registry had durable polymers and were analyzed as the DP-DES group: 1. Xience (Abbott Vascular, Santa Clara, CA, USA), a cobalt chromium everolimus eluting stent (CoCr-EES) with strut thickness 81 μm. (*n* = 99) 2. Resolute Onyx (Medtronic, Santa Rosa, CA, USA), a zotarolimus eluting stent (ZES) with strut thickness 81 μm. (*n* = 61) 3. Endeavor (Medtronic, Santa Rosa, CA, USA), a ZES with strut thickness 91 μm. (*n* = 32) 4. Promus (Boston Scientific, Marlborough, MA, USA), a platinum chromium everolimus eluting stent (PtCr-EES) with strut thickness 81 μm (*n* = 80).

Meanwhile, the following stents in the ROCK registry had biodegradable polymers and were analyzed as the DP-DES group: 1. Ultimaster (Terumo, Tokyo, Japan), a cobalt chromium sirolimus eluting stent (CoCr-SES) with strut thickness 80 μm. (*n* = 44) 2. Orsiro (Biotronik, Berlin, Germany), a CoCr-SES with strut thickness 60 μm for stent diameter 2.25–3.0 mm and 80 μm for larger diameters. (*n* = 31) 3. Synergy (Boston Scientific, Marlborough, MA, USA), a platinum chromium everolimus eluting stent (PtCr-EES) with strut thickness 74 μm. (*n* = 150) 4. Nobori (Terumo, Tokyo, Japan), a stainless steel biolimus eluting stent (BES) with strut thickness 112 μm. (*n* = 6) 5. Biomatrix (Biosensors International, Morges, Switzerland), a CoCr-BES with strut thickness 83 μm (*n* = 7).

### 2.3. Clinical Outcomes and Definition

The primary clinical outcome of this study was all-cause death during 3 years of follow-up. The secondary outcomes were 3-year cardiovascular death, and target-lesion failure (TLF) defined as a composite of cardiac death (CD), target-vessel spontaneous myocardial infarction (TVMI), or ischemia-driven target-lesion revascularization (TLR).

Technical success was defined as residual stenosis of less than 30% in the presence of grade III Thrombolysis in Myocardial Infarction (TIMI) flow [18]. Procedural success was defined as technical success without in-hospital major adverse cerebral and cardiac events, including in-hospital death, in-hospital cerebrovascular accident (CVA), urgent revascularization (PCI or surgery) following the index procedure, procedure-related atrioventricular block requiring temporary pacemaker insertion, type D-F coronary perforation or dissection, intervention (including surgery) due to cardiac tamponade, and peri-procedure MI. Target-vessel spontaneous MI was defined as spontaneous MI which could be clearly attributed to the target vessel. Spontaneous MI was defined as a creatine kinase-myocardial band (CK-MB), or troponin increase above normal range with signs or symptoms of ischemia at any time during follow-up after discharge. Peri-procedural MI was defined as a CK-MB peak elevation of over 10-fold above the upper normal range occurring within 48 h after the index procedure. TLR was defined as any revascularization (PCI or surgery) of the treated lesion. Bleeding events were defined according to the TIMI bleeding criteria [18]. All clinical events were confirmed by source documentation collected at each hospital and centrally adjudicated by an independent group of clinicians unaware of the procedural details.

### 2.4. Statistical Analysis

Continuous variables are presented as the mean ± standard deviation or median and interquartile range and compared using the Student’s *t*-test or Mann–Whitney U-test, as appropriate. Categorical variables are presented as numbers and percentages and compared using the chi-square test or Fisher’s exact test. Clinical endpoints were compared using event curves were constructed using the Kaplan–Meier method and compared using the log-rank *p*-value. To adjust for confounding factors, two analyses were performed. First, propensity-matched cohorts were constructed using 1:1 matching on propensity scores obtained from logistic regression and a nearest-neighbor method with a caliper width of 0.2. The covariates in the propensity score were age, sex, previous history of hypertension, diabetes mellitus (DM), chronic kidney disease (CKD), dialysis, previous PCI, stroke, atrial fibrillation, systolic and diastolic blood pressure at admission, left ventricular ejection fraction (LVEF), peak CK-MB, low-density lipoprotein (LDL) cholesterol, HbA1c, clinical diagnosis, number of coronary arteries with stenosis > 50%, lesion location, mean stent diameter, total stent length, total number of stents, and treatment with aspirin or P2Y12 inhibitors. Covariate balance was assessed with a standardized mean difference < 0.1 indicating appropriate balance [19]. Second, univariate Cox regression analysis was used to identify predictors of mortality on all variables listed in Table 1, and multivariate Cox regression analysis by stepwise selection method was performed to identify independent predictors of death on each variable with *p* < 0.1 in univariate analysis. To avoid overfitting, the number of covariates were chosen such that there was 1 predictor variable per 10 events [20]. The relative effect of predictor variables on clinical outcomes were expressed using hazard ratios (HR) and 95% confidence intervals (CI). A two-sided *p*-value < 0.05 was considered statistically significant. All statistical analyses were performed using R Statistical Software version 4.2.1 (R Foundation for Statistical Computing, Vienna, Austria).

## 3. Results

### 3.1. Baseline Characteristics of the Study Population

Table 1 shows the baseline demographic and laboratory characteristics of patients classified according to DES polymer durability. In total, 272 patients underwent PCI with DP-DES and 238 patients with BP-DES. Patients who were implanted BP-DES were more likely to be male, had lower peak CK-MB levels, and were more likely to be treated using IVUS. Procedural success rates were 97.4% and 97.5% for DP-DES and BP-DES, respectively.

Propensity score matching identified 201 matched pairs for analysis. There was no significant difference in baseline characteristics between the two matched groups. After propensity score matching, a decrease in standardized mean difference for all major variables was observed, and only treatment with P2Y12 inhibitors had a standardized mean difference above 0.1, indicating appropriate covariate balance.

### 3.2. Clinical Outcomes According to Polymer Durability

**Figure 2:**
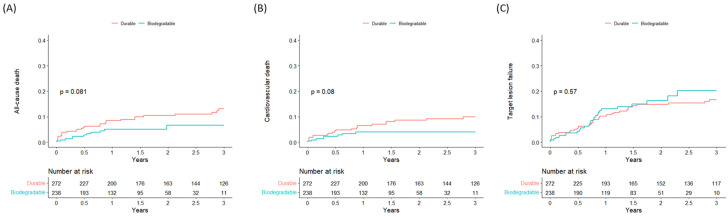
Kaplan–Meier curves of clinical outcomes according to polymer durability. (**A**) all-cause death, (**B**) cardiovascular death, (**C**) target-lesion failure.

The Kaplan–Meier event curves for all-cause mortality, cardiovascular mortality, and TLF are shown in Figure 2. PCI using BP-DES showed a tendency decreased all-cause death compared to DP-DES, and most of the mortality difference occurred within one year of index PCI, although the results were not significant (log-rank *p* = 0.081). Meanwhile, there was a tendency toward decreased cardiovascular death in the BP-DES group which did not reach statistical significance, and there was no difference in TLF between the two groups.

A summary of clinical outcomes according to DES polymer durability is presented in Table 2. In the DP-DES group 29 (10.7%) deaths occurred, and in the BP-DES group 11 (4.7%) deaths occurred. After adjusting for baseline variables using propensity score matching, PCI with BP-DES was associated with a significant decrease for the primary outcome of all-cause death (HR 0.414, CI 0.174–0.988). BP-DES was also associated with a lower risk of cardiovascular death (HR 0.281, CI 0.094–0.843), but there was no difference in TLF (HR 1.048, CI 0.590–1.861) between the two groups.

Univariate Cox regression analysis was done for all the clinical variables listed as baseline characteristics in Table 1, and the results are shown in Table 3. Older age, lower BMI, previous history of dyslipidemia and CVA, lower LVEF, lower Hb, higher peak CK-MB, a diagnosis of NSTEMI or silent ischemia, and a smaller stent diameter were potential factors for increased all-cause mortality. Treatment with aspirin, P2Y12 inhibitors, beta-blockers, and statins were associated with decreased mortality. After multivariate Cox regression, PCI with BP-DES was identified as an independent factor predicting decreased all-cause mortality (HR 0.458, CI 0.224–0.940). Older age, previous history of CVA, lower LVEF, higher peak CK-MB, and no treatment with P2Y12 inhibitors were other factors showing the strongest association with increased all-cause mortality.

## 4. Discussion

BP-DES, compared to DP-DES, was associated with decreased three-year all-cause mortality in patients who underwent PCI with rotational atherectomy. This finding was significant after modifying for confounders using multivariate Cox regression analysis and propensity score matching. PCI with BP-DES also showed better outcomes for cardiovascular death, while there was no difference observed for TLF.

In our study, 510 patients received SGDES implantation for PCI after RA and were followed up for a duration of median 16.1 months. All-cause death occurred at an overall incidence of 10.4%, cardiovascular death 6.8%, and TLF at 14.5%. Our mortality data is comparable with other reports of PCI using RA and DES and indicates high standards of PCI using both DP-DES and BP-DES. Okai et al. reported incidences of cardiac death 10.9% and TVR 21.4% during a median period of 3.8 years of follow-up [21]. Abdel-Wahab et al. reported all-cause death at 4.4%, MI at 3.4%, TVR in 9.9%, and TLR in 6.8% during a follow-up period of median 15 months [9].

Contemporary SGDES are an improvement on the first-generation DES with respect to the antiproliferative agent, polymer biocompatibility, and thinner stent platforms, and have been proven to be superior to BMS or first-generation DES [22,23,24]. Initially there was concern that these improvements may not be true for severe calcified lesions, as the calcified plaques might limit stent expansion so that the thinner struts of the SGDES would be less effective than the thicker first-generation DES [25,26]. However, recent studies conclude that SGDES were indeed more effective even for calcified lesions [27].

Stent polymers have been implicated in the development of restenosis and late and very late stent thrombosis, due to local hypersensitivity and inflammatory reactions [28]. This has led to the development of DES with biodegradable polymers, where only the bare metal platform is left behind after the drug-eluting polymer is degraded. There is accumulating evidence that newer ultrathin stents and biodegradable polymers are associated with more favorable results in patients undergoing PCI compared to thicker strut SGDES and durable polymers. In post hoc analyses of the BIO-RESORT trial, Synergy EES (strut thickness 74 μm) were associated with lower rates of TLR compared to Resolute Integrity ZES (strut thickness 91 μm) in small coronary arteries [13] and calcified lesions [14]. In the BIOFLOW V [15] and BIOSTEMI [16] trials, superior outcomes were observed with Orsiro CoCr-SES-BP (strut thickness 60/80 μm) compared to Xience CoCr-EES-DP. Our study is consistent with previous studies demonstrating improved clinical outcomes with BP-DES and extends the results to the population of patients undergoing RA for severely calcified coronary artery lesions.

An alternative explanation for the superior performance of BP-DES is that the newer stents have thinner strut platforms compared to older DP-DES. Stents with ultrathin struts on the order of 60 μm have been associated with decreased TLF and TVMI [29], whereas older BP-DES with thicker struts have been at best non-inferior to contemporary DP-DES [30,31]. The heterogenous composition of both DP-DES and especially BP-DES in our study, while a limitation, is also a strength since we incorporated stents with thicker struts as well as Orsiro SES-BP, meaning that the effects observed in our analysis is more likely to be due to polymer durability than from strut design.

The effects of DES in conjunction after the vessel injury caused by RA are yet unclear. Theoretically, as the lumen left after RA is relatively smooth and free of calcifications, this would manifest as improved outcomes after RA and DES compared to DES alone [4]. RA has also been shown to decrease polymer damage during stent delivery through calcified lesions [32]. Yet, in the ROTAXUS trial, RA + DES was not associated with improved clinical outcomes, as the acute gains from RA were offset by increased late lumen loss, possibly as a result of vessel injury sustained during the RA procedure [33]. Vessel injury may also lead to increased inflammation, creating an environment where BP-DES may be potentially superior to DP-DES. A recent study by Mankerious et al. reported that Orsiro CoCr-SES-BP was associated with lower rates of TLF in small vessels after PCI using RA compared to EES-DP, although this effect was not significant when larger vessels were also considered [34]. Our analysis also suggests that BP-DES is associated with superior outcomes compared to DP-DES when used after RA. Further studies are needed to ascertain the effect of BP-DES in the RA population.

This study has several limitations. First, this was not a randomized trial but a retrospective study and may be subject to inherent biases, although we used propensity score matching and multivariate Cox regression analysis to eliminate confounding variables. A selection bias cannot be excluded due to the heterogeneous composition of both BP-DES and DP-DES groups, and the lack of clear and definite indications for choosing BP-DES or DP-DES. Second, the number of patients in the registry was not large, and our study may have lacked sufficient power to distinguish between outcomes or to incorporate more variables into the multivariate regression analysis. However, since in contemporary practice RA is generally reserved for the most severely calcified lesions unamenable to high pressure balloons, the number of patients undergoing PCI using RA cannot be very large, and the number of patients in our registry is similar to other studies on RA [9,21,34]. Third, the difference in the primary outcome of all-cause death reached only borderline significance after adjustment with propensity score matching, and there is a possibility that the results were only due to chance. Fourth, stent characteristics other than polymer durability may have been confounding variables that were unaccounted for in our analysis.

## 5. Conclusions

BP-DES was associated with decreased all-cause mortality and cardiovascular mortality compared to DP-DES in patients who underwent PCI using RA for calcified coronary lesions. There was no difference in TLF between the two groups. However, these results should only be considered as hypothesis generating, and further studies are needed to confirm these results.

## Figures and Tables

**Table 1:** Clinical, laboratory, and angiographic characteristics according to stent polymer composition.

	Before PS Matching					After PS Matching				
Characteristics	DP-DES (*n* = 272)	BP-DES (*n* = 238)	*p*-Value		SMD	DP-DES (*n* = 201)	BP-DES (*n* = 201)	*p*-Value		SMD
Demographic and clinical characteristics										
Age (years)	74 (66, 78)	73 (64, 79)	0.373		0.084	74 (66, 78)	73 (64, 79)	0.636		0.062
Sex (Male, %)	150 (55.1)	156 (65.5)	0.021		0.214	127 (63.2)	126 (62.7)	1.000		0.010
BMI (kg/m^2^)	23.7 (21.7, 26.1)	24.0 (22.1, 27.0)	0.291		0.051	23.8 (22.5, 25.7)	23.8 (22.2, 26.9)	0.780		0.021
HTN (%)	216 (79.4)	183 (76.9)	0.561		0.061	159 (79.1)	159 (79.1)	1.000		<0.001
DM (%)	146 (53.7)	141 (59.2)	0.240		0.112	116 (57.7)	118 (58.7)	0.919		0.020
Smoking (%)	48 (17.6)	51 (21.4)	0.335		0.095	38 (18.9)	42 (20.9)	0.708		0.050
Dyslipidemia (%)	125 (46.0)	104 (43.7)	0.673		0.045	90 (44.8)	90 (44.8)	1.000		<0.001
CKD (%)	48 (17.6)	50 (21.0)	0.396		0.085	34 (16.9)	34 (16.9)	1.000		<0.001
Dialysis (%)	25 (9.2)	27 (11.3)	0.512		0.071	16 (8.0)	17 (8.5)	1.000		0.018
Previous PCI (%)	68 (25.0)	66 (27.7)	0.550		0.062	56 (27.9)	49 (24.4)	0.496		0.079
Previous MI (%)	29 (10.7)	33 (13.9)	0.333		0.098	26 (12.9)	24 (11.9)	0.88		0.030
Previous CVA (%)	33 (12.1)	29 (12.2)	1.000		0.002	27 (13.4)	26 (12.9)	1.000		0.015
Atrial fibrillation (%)	26 (9.6)	21 (8.8)	0.894		0.025	19 (9.5)	19 (9.5)	1.000		<0.001
SBP (mmHg)	130 (120, 140)	130 (120, 145)	0.310		0.065	130 (120, 140)	129 (120, 140)	0.797		0.019
DBP (mmHg)	77 (67, 80)	74 (66, 80)	0.844		0.029	77 (66, 80)	74 (66, 80)	0.888		0.065
Laboratory findings										
LVEF (%)	57.0 (46.0, 63.0)	57.0 (42.0, 63.0)	0.123		0.138	57.0 (45.0, 62.0)	57.0 (42.0, 63.0)	0.950		0.041
Hb (mg/dL)	12.1 ±1.8	12.4 ± 1.9	0.122		0.137	12.2 ± 1.8	12.4 ± 1.9	0.350		0.093
Platelet (10^9^/L)	223 ± 70.9	218 ± 72.8	0.491		0.061	210 (170, 248)	213 (165, 264)	0.526		0.066
HbA1c (%)	6.3 (5.7, 7.3)	6.3 (5.7, 7.1)	0.782		0.048	6.4 (5.7, 7.2)	6.2 (5.7, 7.1)	0.675		0.012
LDL-cholesterol (mg/dL)	83.0 (63.2, 100.5)	75.1 (59.2, 101.1)	0.229		0.061	79.0 (61.0, 96.7)	79.0 (60.5, 102.2)	0.723		0.059
Initial Cr (mg/dL)	0.90 (0.73, 1.22)	0.93 (0.80, 1.22)	0.043		0.216	0.91 (0.74, 1.28)	0.91 (0.78, 1.14)	0.794		0.008
Peak CK-MB (ng/mL)	6.90 (3.10, 19.30)	3.86 (1.90, 12.52)	<0.001		0.072	6.96 (3.30, 18.83)	4.12 (2.02, 15.38)	0.011		0.011
Angiographic and procedural characteristics										
Diagnosis (%)			0.607		0.146			0.998		0.034
SA	94 (34.6)	83 (34.9)				68 (33.8)	68 (33.8)			
UA	90 (33.1)	70 (29.4)				66 (32.8)	65 (32.3)			
NSTEMI	66 (24.3)	57 (23.9)				45 (22.4)	46 (22.9)			
STEMI	9 (3.3)	9 (3.8)				9 (4.5)	8 (4.0)			
Silent ischemia	13 (4.8)	19 (8.0)				13 (6.5)	14 (7.0)			
Coronary artery (%)			0.545		0.097			0.705		0.083
LAD	188 (69.1)	159 (66.8)				140 (69.7)	133 (66.2)			
LCX	19 (7.0)	23 (9.7)				17 (8.5)	21 (10.4)			
RCA	65 (23.9)	56 (23.5)				44 (21.9)	47 (23.4)			
Multivessel disease (%)	107 (84.3)	137 (76.5)	0.131		0.195	182 (90.5)	177 (88.1)	0.519		0.081
LM disease (%)	42 (15.4)	36 (15.1)	1.000		0.009	35 (17.4)	31 (15.4)	0.686		0.054
Stent (%)										
BES-BP	0 (0.0)	12 (5.1)				0 (0.0)	11 (5.5)			
CoCr-SES-BP	0 (0.0)	75 (31.6)				0 (0.0)	65 (32.5)			
PtCr-EES-BP	0 (0.0)	150 (63.3)				0 (0.0)	124 (62.0)			
CoCr-EES-DP	99 (36.4)	0 (0.0)				75 (37.3)	0 (0.0)			
PtCr-EES-DP	80 (29.4)	0 (0.0)				54 (26.9)	0 (0.0)			
ZES-DP	93 (34.2)	0 (0.0)				72 (35.8)	0 (0.0)			
Mean stent diam. (mm)	3.00 (2.75, 3.25)	2.92 (2.75, 3.23)	0.640		0.018	3.00 (2.75, 3.28)	2.89 (2.75, 3.23)	0.595		0.033
Total stent length (mm)	46 (34, 61)	51 (33, 66)	0.222		0.085	46 (38, 60)	51 (33, 66)	0.528		0.028
Number of stents	2 (1, 2)	2 (1, 2)	0.865		0.063	2 (1, 2)	2 (1, 2)	0.734		0.075
Max. burr diam. (mm)	1.50 (1.50, 1.50)	1.50 (1.25, 1.50)	0.609		0.038	1.50 (1.50, 1.50)	1.50 (1.25, 1.50)	0.612		0.037
IVUS (%)	111 (40.8)	135 (56.7)	<0.001		0.322	100 (49.8)	108 (53.7)	0.485		0.080
Medication at discharge										
Aspirin (%)	265 (98.1)	234 (98.3)	1.000	†	0.013	198 (98.5)	197 (98.0)	1.000	†	0.038
P2Y12 inhibitor (%)	266 (98.5)	237 (99.6)	0.378	†	0.110	199 (99.0)	201 (100.0)	0.499	†	0.142
RASB (%)	198 (73.3)	170 (71.4)	0.704		0.148	144 (71.6)	142 (70.6)	0.912		0.022
BB (%)	176 (65.2)	138 (58.0)	0.115		0.043	125 (62.2)	121 (60.2)	0.759		0.041
Statin (%)	246 (91.8)	227 (95.4)	0.147		0.147	187 (93.0)	190 (94.5)	0.680		0.062

Continuous data are presented as mean ± standard deviation for normally distributed variables, and as median (Q1, Q3) for non-normally distributed variables. † Variables compared using Fisher’s exact test. PS, propensity score; BP-DES, biodegradable polymer drug eluting stent; DP-DES, durable polymer drug eluting stent; BMI, body mass index; HTN, hypertension; DM, diabetes mellitus; CAD, coronary artery disease; CKD, chronic kidney disease; PCI, percutaneous coronary intervention; MI, myocardial infarction; CVA, cerebrovascular accident; SBP, systolic blood pressure; DBP; diastolic blood pressure; LVEF, left ventricular ejection fraction; Hb, hemoglobin; HbA1C, glycated hemoglobin; LDL, low density lipoprotein; Cr, creatinine; CK-MB, creatinine kinase myocardial band; SA, stable angina; UA, unstable angina; NSTEMI, non-ST elevation myocardial infarction; STEMI, ST elevation myocardial infarction; LAD, left anterior descending; LCX, left circumflex; RCA, right coronary artery; LM, left main; CoCr-SES, cobalt chromium sirolimus eluting stent; PtCR-EES, platinum chromium everolimus eluting stent; CoCr-EES, cobalt chromium everolimus eluting stent; ZES, zotarolimus eluting stent; diam, diameter; IVUS, intravascular ultrasound; RASB, renin-angiotensin system blocker; BB, beta blocker.

**Table 2:** Clinical endpoints before and after adjustment using propensity score matching.

	DP-DES *n* (%)	BP-DES *n* (%)	Crude		PSM	
			HR (CI)	*p*-Value	HR (CI)	*p*-Value
All-cause death	29 (10.7)	11 (4.7)	0.539 (0.267–1.090)	0.085	0.414 (0.174–0.988)	0.047
Cardiovascular death	22 (8.1)	8 (3.4)	0.490 (0.217–1.108)	0.087	0.281 (0.094–0.843)	0.024
Target lesion failure	37 (13.6)	29 (12.2)	1.155 (0.705–1.891)	0.567	1.048 (0.590–1.861)	0.874

DP-DES, durable polymer drug eluting stent; BP-DES, biodegradable polymer drug eluting stent; PSM, propensity score matching.

**Table 3:** Univariate and multivariate Cox regression analysis on all-cause mortality.

	Univariate			Multivariate	
Characteristics	HR (CI)	*p*-Value		HR (CI)	*p*-Value
Polymer		0.085	*		0.033
DP-DES	reference			reference	
BP-DES	0.539 (0.267–1.090)			0.458 (0.224–0.940)	
Age	1.054 (1.019–1.089)	0.002	*	1.048 (1.008–1.088)	0.017
Sex (Male)	0.794 (0.448–1.408)	0.430			
BMI	0.818 (0.741–0.903)	<0.001	*		
HTN	0.838 (0.435–1.614)	0.597			
DM	1.165 (0.650–2.086)	0.608			
Smoking	0.949 (0.459–1.963)	0.888			
Dyslipidemia	0.521 (0.282–0.963)	0.037	*		
CKD	1.276 (0.634–2.571)	0.495			
Dialysis	0.679 (0.211–2.187)	0.516			
Previous PCI	0.788 (0.401–1.548)	0.488			
Previous MI	0.887 (0.351–2.242)	0.801			
Previous CVA	2.705 (1.427–5.127)	0.002	*	2.656 (1.294–5.451)	0.008
Atrial fibrillation	1.606 (0.719–3.585)	0.248			
SBP	0.999 (0.986–1.011)	0.820			
DBP	1.001 (0.976–1.024)	0.981			
LVEF	0.965 (0.947–0.984)	<0.001	*	0.966 (0.945–0.987)	0.002
Hb	0.736 (0.626–0.866)	<0.001	*		
Platelet	1.001 (0.997–1.005)	0.773			
HbA1c	1.089 (0.878–1.349)	0.438			
LDL-cholesterol	1.002 (0.994–1.009)	0.649			
Initial Cr	0.923 (0.766–1.112)	0.397			
Peak CK-MB	1.008 (1.003–1.013)	<0.001	*	1.007 (1.001–1.012)	0.012
Diagnosis					
SA	reference		*		
UA	2.610 (0.980–6.955)	0.055			
NSTEMI	6.253 (2.546–15.385)	<0.001			
STEMI	1.928 (0.232–16.016)	0.543			
Silent Ischemia	5.601 (1.708–18.369)	0.004			
Coronary artery					
LAD	reference				
LCX	0.861 (0.304–2.436)	0.778			
RCA	0.885 (0.446–1.757)	0.727			
Multivessel disease	1.654 (0.495–5.528)	0.414			
LM disease	0.907 (0.406–2.025)	0.812			
Mean stent diameter	0.363 (0.145–0.911)	0.031	*		
Number of stents	1.168 (0.921–1.481)	0.202			
Total stent length	1.005 (0.996–1.013)	0.255			
Max. burr diameter	0.415 (0.108–2.215)	0.303			
IVUS	0.573 (0.313–1.049)	0.071	*		
Aspirin	0.212 (0.066–0.685)	0.010	*		
P2Y12 inhibitor	0.039 (0.014–0.109)	<0.001	*	0.011 (0.003–0.042)	<0.001
RASB	0.733 (0.408–1.315)	0.297			
BB	0.383 (0.214–0.685)	0.001	*		
Statin	0.243 (0.113–0.521)	<0.001	*		

* indicates variables that were used in multivariate regression. BP-DES, biodegradable polymer drug eluting stent; DP-DES, durable polymer drug eluting stent; BMI, body mass index; HTN, hypertension; DM, diabetes mellitus; CAD, coronary artery disease; CKD, chronic kidney disease; PCI, percutaneous coronary intervention; MI, myocardial infarction; CVA, cerebrovascular accident; SBP, systolic blood pressure; DBP; diastolic blood pressure; LVEF, left ventricular ejection fraction; Hb, hemoglobin; HbA1C, glycated hemoglobin; LDL, low density lipoprotein; Cr, creatinine; CK-MB, creatinine kinase myocardial band; SA, stable angina; UA, unstable angina; NSTEMI, non-ST elevation myocardial infarction; STEMI, ST elevation myocardial infarction; LAD, left anterior descending; LCX, left circumflex; RCA, right coronary artery; LM, left main; BP, biodegradable polymer; DP, durable polymer; CoCr-SES, cobalt chromium sirolimus eluting stent; PtCR-EES, platinum chromium everolimus eluting stent; CoCr-EES, cobalt chromium everolimus eluting stent; ZES, zotarolimus eluting stent; IVUS, intravascular ultrasound; RASB, renin-angiotensin system blocker; BB, beta blocker, NOAC, novel oral anticoagulants.

## Data Availability

Data are available from the corresponding author on reasonable request.
